# Ethyl (1*R*,4*S*,5*R*,9*S*,10*R*,13*S*)-5,9,13-trimethyl-14-methyl­ene-14-oxotetra­cyclo­[11.2.1.0^1,10^.0^4,9^]hexa­decane-5-carboxyl­ate

**DOI:** 10.1107/S1600536809053380

**Published:** 2009-12-16

**Authors:** Hao Shi

**Affiliations:** a The College of Pharmaceutical Science, Zhejiang University of Technology, Hangzhou 310014, People’s Republic of China

## Abstract

The title compound, C_22_H_34_O_3_, was synthesized from isosteviol. The asymmetric unit contains of two independent mol­ecules with the same absolute configurations. In both the mol­ecules, the three six-membered rings adopt chair conformations, the stereochemistry of the *A*/*B* and *B*/*C* ring junctions are *trans*, and the five-membered ring *D* adopts an envelope conformation.

## Related literature

Since the title compound was prepared from isosteviol, the configuration can be deduced from the known chirality of the isosteviol, see: Rodrigues & Lechat (1988 ▶), Xue *et al.* (1993 ▶). For the pharmacological activity of isosteviol, see: Liu *et al.* (2001 ▶); Mizushina *et al.* (2005 ▶); Wong *et al.* (2004 ▶); Zhang & Xu (2004 ▶). For ring conformations, see: Cremer & Pople (1975 ▶). 
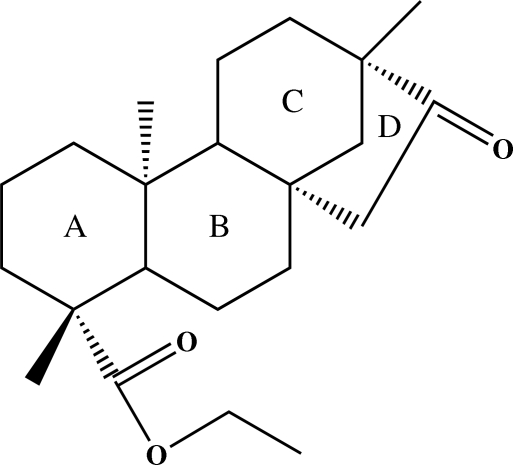


## Experimental

### 

#### Crystal data

C_22_H_34_O_3_
*M* *_r_* = 346.49
Triclinic, 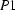

*a* = 6.5365 (9) Å
*b* = 12.5639 (15) Å
*c* = 13.1212 (16) Å
α = 69.174 (1)°
β = 87.518 (2)°
γ = 79.018 (1)°
*V* = 988.4 (2) Å^3^
*Z* = 2
Mo *K*α radiation
μ = 0.08 mm^−1^
*T* = 293 K
0.49 × 0.42 × 0.15 mm

#### Data collection

Bruker SMART CCD area-detector diffractometer
Absorption correction: multi-scan (*SADABS*; Bruker, 1999 ▶) *T*_min_ = 0.964, *T*_max_ = 0.989
5226 measured reflections
3451 independent reflections
1824 reflections with *I* > 2σ(*I*)
*R*_int_ = 0.034

#### Refinement

*R*[*F* ^2^ > 2σ(*F* ^2^)] = 0.067
*wR*(*F* ^2^) = 0.189
*S* = 0.90
3451 reflections
459 parameters
3 restraints
H-atom parameters constrained
Δρ_max_ = 0.26 e Å^−3^
Δρ_min_ = −0.27 e Å^−3^

### 

Data collection: *SMART* (Bruker, 1999 ▶); cell refinement: *SAINT* (Bruker, 1999 ▶); data reduction: *SAINT*; program(s) used to solve structure: *SHELXS97* (Sheldrick, 2008 ▶); program(s) used to refine structure: *SHELXL97* (Sheldrick, 2008 ▶); molecular graphics: *ORTEP-3* (Farrugia, 1997 ▶); software used to prepare material for publication: *SHELXL97* nd *PLATON* (Spek, 2009 ▶).

## Supplementary Material

Crystal structure: contains datablocks I, global. DOI: 10.1107/S1600536809053380/dn2514sup1.cif

Structure factors: contains datablocks I. DOI: 10.1107/S1600536809053380/dn2514Isup2.hkl

Additional supplementary materials:  crystallographic information; 3D view; checkCIF report

## Figures and Tables

**Table 1:** Comparison of the puckering parameters (Å, °) for the six and five-membered rings in mol­ecules 1 and 2

Mol­ecule		1			2	
Puckering parameters	*Q*	θ	ϕ	*Q*	θ	ϕ
Ring *A*1, *A*2	0.536 (7)	180.0 (7)	273 (27)	0.545 (8)	177.2 (8)	251 (29)
Ring *B*1, *B*2	0.557 (7)	9.5 (7)	72 (4)	0.561 (7)	7.8 (7)	71 (5)
Ring *C*1, *C*2	0.633 (7)	164.8 (6)	120 (3)	0.646 (8)	163.2 (7)	131 (2)
Puckering parameters		q_2_	ϕ_2_		q_2_	ϕ_2_
Ring *D*1, *D*2		0.467 (8)	333.7 (10)		0.452 (8)	332.2 (11)

Ring *A*1 atoms C2–C7, *A*2 C24–C29, *B*1 C6–C11, *B*2 C28–C33, *C*1 C10–C14/C17, *C*2 C32–C36/C39, *D*1 C14–C16/C10/C17 and *D*2 C36–C38/C32/C39.

**Fig. 1.**
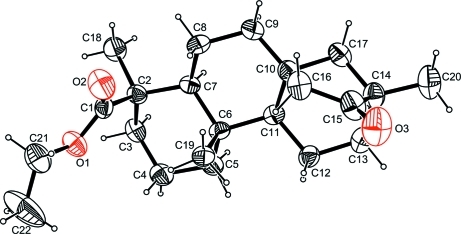
Molecular view of molecule 1 with the atom labeling scheme. Ellipsoids are drawn at the 30% probability level. H atoms are represented as small spheres of arbitrary radii.

**Fig. 2.**
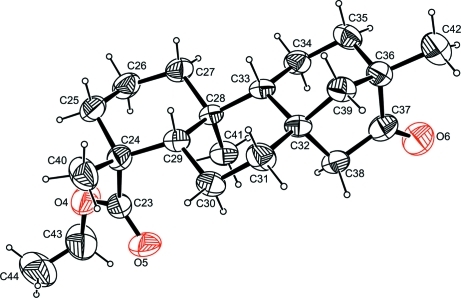
Molecular view of molecule 2 with the atom labeling scheme. Ellipsoids are drawn at the 30% probability level. H atoms are represented as small spheres of arbitrary radii.

**Fig. 3.**
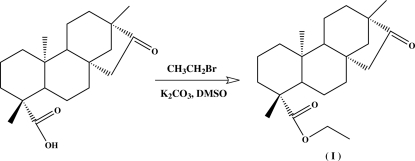
The formation of the title compound

## supplementary crystallographic information

### Comment

Since the natural diterpenoid isosteviol has good pharmacological activity against a number of significant diseases including ischemia–reperfusion injury, hypertension, and cancer (Liu, *et al.*, 2001; Mizushina *et al.*, 2005; Wong *et al.*, 2004; Zhang & Xu, 2004), the title compound was derived from it. Two independent molecules with identical absolute configuration are present in the asymmetric unit . Both molecule 1 (Fig.1) and molecule 2 (Fig.2) are built up from three six-membered rings and one five-membered ring. Some geometrical features (puckering parameters, Cremer & Pople, 1975) of these rings were investigated (Table 1) using *PLATON* (Spek, 2009).

Since the title compound was prepared from isosteviol, the configuration can be deduced from the known chirality of the isosteviol (Rodrigues & Lechat, 1988; Xue *et al.*, 1993), and thus Fig. 1 and Fig. 2 represents the correct absolute configuration.

### Experimental

A mixture of isosteviol (1 mmol), K_2_CO_3_ (2 mmol) and 10 mL of DMSO was transferred into a flask connected with refluxing equipment. After stirring for 5 min, bromoethane (1.2 mmol) was added, then stirring at 40°C for 6 h, the reaction mixture was poured into 40 mL water, extracted with 3×30 mL e thyl acetate, the ethyl acetate layer was washed with 3×30 mL water, then dryed with anhydrous Na_2_SO_4_, purified with silion colum and the title compound was gained. Crystals suitable for X-ray structure analysis were obtained by slow evaporation from a solution of isopropyl alcohol at room temperature.

### Refinement

All H atoms were placed in geometrical positions and constrained to ride on their parent atoms with C–H distances in the range 0.96–0.98 Å, They were treated as riding atoms, with *U*_iso_(H) = 1.5Ueq(C) for methyl H and 1.2Ueq(C) for other H atoms). Friedel reflections were merged before the final refinement and the stereochemistry is added according to the starting material.

### Crystal data

**Table:**

C_22_H_34_O_3_	*Z* = 2
*M**_r_* = 346.49	*F*(000) = 380
Triclinic, *P*1	*D*_x_ = 1.164 Mg m^−^^3^
Hall symbol: P 1	Mo *K*α radiation, λ = 0.71073 Å
*a* = 6.5365 (9) Å	Cell parameters from 1056 reflections
*b* = 12.5639 (15) Å	θ = 3.2–19.4°
*c* = 13.1212 (16) Å	µ = 0.08 mm^−^^1^
α = 69.174 (1)°	*T* = 293 K
β = 87.518 (2)°	Plate, colorless
γ = 79.018 (1)°	0.49 × 0.42 × 0.15 mm
*V* = 988.4 (2) Å^3^	

### Data collection

**Table:**

Bruker SMART CCD area-detector diffractometer	3451 independent reflections
Radiation source: fine-focus sealed tube	1824 reflections with *I* > 2σ(*I*)
graphite	*R*_int_ = 0.034
φ and ω scans	θ_max_ = 25.0°, θ_min_ = 1.7°
Absorption correction: multi-scan (*SADABS*; Bruker, 1999)	*h* = −7→5
*T*_min_ = 0.964, *T*_max_ = 0.989	*k* = −14→14
5226 measured reflections	*l* = −14→15

### Refinement

**Table:**

Refinement on *F*^2^	Primary atom site location: structure-invariant direct methods
Least-squares matrix: full	Secondary atom site location: difference Fourier map
*R*[*F*^2^ > 2σ(*F*^2^)] = 0.067	Hydrogen site location: inferred from neighbouring sites
*wR*(*F*^2^) = 0.189	H-atom parameters constrained
*S* = 0.90	*w* = 1/[σ^2^(*F*_o_^2^) + (0.1087*P*)^2^] where *P* = (*F*_o_^2^ + 2*F*_c_^2^)/3
3451 reflections	(Δ/σ)_max_ = 0.001
459 parameters	Δρ_max_ = 0.26 e Å^−^^3^
3 restraints	Δρ_min_ = −0.27 e Å^−^^3^

### Special details

**Table:**

Geometry. All e.s.d.'s (except the e.s.d. in the dihedral angle between two l.s. planes) are estimated using the full covariance matrix. The cell e.s.d.'s are taken into account individually in the estimation of e.s.d.'s in distances, angles and torsion angles; correlations between e.s.d.'s in cell parameters are only used when they are defined by crystal symmetry. An approximate (isotropic) treatment of cell e.s.d.'s is used for estimating e.s.d.'s involving l.s. planes.
Refinement. Refinement of *F*^2^ against ALL reflections. The weighted *R*-factor *wR* and goodness of fit *S* are based on *F*^2^, conventional *R*-factors *R* are based on *F*, with *F* set to zero for negative *F*^2^. The threshold expression of *F*^2^ > σ(*F*^2^) is used only for calculating *R*-factors(gt) *etc*. and is not relevant to the choice of reflections for refinement. *R*-factors based on *F*^2^ are statistically about twice as large as those based on *F*, and *R*- factors based on ALL data will be even larger.

### Fractional atomic coordinates and isotropic or equivalent isotropic displacement parameters (Å^2^)

**Table:**

	*x*	*y*	*z*	*U*_iso_*/*U*_eq_	
O1	0.5517 (6)	0.8839 (4)	0.5497 (4)	0.0762 (13)	
O2	0.4744 (8)	0.7550 (4)	0.7036 (4)	0.0915 (16)	
O3	0.3864 (9)	0.1982 (5)	0.6485 (6)	0.111 (2)	
O4	0.5700 (8)	0.2697 (5)	0.1516 (6)	0.1072 (19)	
O5	0.6802 (9)	0.3579 (5)	0.2518 (5)	0.1071 (19)	
O6	0.7199 (9)	0.9816 (6)	0.0024 (6)	0.120 (2)	
C1	0.5924 (9)	0.7852 (5)	0.6322 (6)	0.0558 (16)	
C2	0.8169 (9)	0.7176 (5)	0.6312 (5)	0.0536 (15)	
C3	0.9253 (9)	0.7692 (5)	0.5244 (5)	0.0617 (17)	
H3A	0.9044	0.8526	0.5052	0.074*	
H3B	1.0739	0.7390	0.5351	0.074*	
C4	0.8461 (11)	0.7427 (6)	0.4310 (5)	0.0693 (18)	
H4A	0.9272	0.7727	0.3671	0.083*	
H4B	0.7021	0.7817	0.4137	0.083*	
C5	0.8601 (10)	0.6134 (5)	0.4585 (5)	0.0616 (16)	
H5A	1.0059	0.5763	0.4656	0.074*	
H5B	0.7987	0.6012	0.3985	0.074*	
C6	0.7511 (9)	0.5552 (5)	0.5634 (5)	0.0525 (15)	
C7	0.8269 (9)	0.5851 (5)	0.6582 (5)	0.0524 (15)	
H7	0.9762	0.5518	0.6666	0.063*	
C8	0.7371 (10)	0.5226 (5)	0.7664 (5)	0.0613 (16)	
H8A	0.5860	0.5421	0.7616	0.074*	
H8B	0.7846	0.5460	0.8228	0.074*	
C9	0.8091 (11)	0.3925 (5)	0.7942 (5)	0.0702 (19)	
H9A	0.9591	0.3741	0.8059	0.084*	
H9B	0.7475	0.3524	0.8623	0.084*	
C10	0.7553 (10)	0.3465 (5)	0.7091 (5)	0.0586 (16)	
C11	0.8168 (9)	0.4196 (5)	0.5961 (5)	0.0536 (15)	
H11	0.9695	0.4042	0.5984	0.064*	
C12	0.7574 (10)	0.3752 (5)	0.5104 (5)	0.0615 (16)	
H12A	0.8222	0.4125	0.4424	0.074*	
H12B	0.6075	0.3959	0.4979	0.074*	
C13	0.8246 (12)	0.2435 (6)	0.5439 (6)	0.077 (2)	
H13A	0.7614	0.2176	0.4936	0.093*	
H13B	0.9747	0.2246	0.5388	0.093*	
C14	0.7626 (10)	0.1792 (5)	0.6594 (6)	0.0686 (18)	
C15	0.5375 (11)	0.2305 (6)	0.6695 (6)	0.077 (2)	
C16	0.5268 (10)	0.3286 (6)	0.7102 (6)	0.0718 (18)	
H16A	0.4734	0.3089	0.7834	0.086*	
H16B	0.4378	0.3981	0.6626	0.086*	
C17	0.8666 (11)	0.2207 (5)	0.7359 (6)	0.0700 (19)	
H17A	1.0145	0.2164	0.7225	0.084*	
H17B	0.8481	0.1747	0.8115	0.084*	
C18	0.9342 (11)	0.7363 (6)	0.7214 (6)	0.076 (2)	
H18A	0.9432	0.8166	0.6999	0.114*	
H18B	1.0720	0.6901	0.7321	0.114*	
H18C	0.8601	0.7137	0.7882	0.114*	
C19	0.5141 (9)	0.5991 (5)	0.5419 (5)	0.0595 (16)	
H19A	0.4674	0.5713	0.4893	0.089*	
H19B	0.4851	0.6824	0.5143	0.089*	
H19C	0.4423	0.5710	0.6088	0.089*	
C20	0.8070 (13)	0.0479 (6)	0.6884 (8)	0.099 (3)	
H20A	0.7698	0.0110	0.7625	0.149*	
H20B	0.9527	0.0220	0.6803	0.149*	
H20C	0.7265	0.0281	0.6406	0.149*	
C21	0.3467 (11)	0.9488 (7)	0.5513 (7)	0.090 (2)	
H21A	0.3459	0.9935	0.5984	0.108*	
H21B	0.2483	0.8969	0.5787	0.108*	
C22	0.2882 (17)	1.0272 (11)	0.4378 (9)	0.168 (6)	
H22A	0.3759	1.0843	0.4148	0.252*	
H22B	0.1453	1.0652	0.4345	0.252*	
H22C	0.3057	0.9830	0.3905	0.252*	
C23	0.5436 (12)	0.3482 (7)	0.2027 (7)	0.081 (2)	
C24	0.3229 (11)	0.4178 (6)	0.1861 (6)	0.0723 (19)	
C25	0.2018 (12)	0.4081 (7)	0.0929 (7)	0.083 (2)	
H25A	0.2179	0.3269	0.1018	0.100*	
H25B	0.0548	0.4370	0.0986	0.100*	
C26	0.2701 (12)	0.4730 (7)	−0.0187 (6)	0.083 (2)	
H26A	0.1830	0.4657	−0.0730	0.099*	
H26B	0.4128	0.4393	−0.0280	0.099*	
C27	0.2558 (11)	0.5991 (6)	−0.0361 (5)	0.075 (2)	
H27A	0.1102	0.6344	−0.0362	0.090*	
H27B	0.3093	0.6371	−0.1073	0.090*	
C28	0.3773 (9)	0.6210 (5)	0.0515 (5)	0.0566 (16)	
C29	0.3104 (10)	0.5473 (5)	0.1658 (5)	0.0648 (18)	
H29	0.1608	0.5774	0.1664	0.078*	
C30	0.4034 (11)	0.5761 (6)	0.2556 (5)	0.0703 (19)	
H30A	0.3623	0.5279	0.3263	0.084*	
H30B	0.5544	0.5600	0.2535	0.084*	
C31	0.3289 (12)	0.7015 (6)	0.2403 (6)	0.076 (2)	
H31A	0.3917	0.7178	0.2976	0.091*	
H31B	0.1792	0.7146	0.2493	0.091*	
C32	0.3777 (10)	0.7881 (5)	0.1293 (5)	0.0619 (17)	
C33	0.3076 (9)	0.7516 (5)	0.0370 (5)	0.0557 (15)	
H33	0.1550	0.7648	0.0387	0.067*	
C34	0.3586 (11)	0.8358 (6)	−0.0715 (5)	0.0726 (19)	
H34A	0.2922	0.8222	−0.1293	0.087*	
H34B	0.5080	0.8208	−0.0809	0.087*	
C35	0.2887 (12)	0.9627 (6)	−0.0830 (6)	0.080 (2)	
H35A	0.3509	1.0110	−0.1469	0.095*	
H35B	0.1385	0.9837	−0.0939	0.095*	
C36	0.3494 (11)	0.9864 (6)	0.0168 (6)	0.075 (2)	
C37	0.5793 (12)	0.9336 (7)	0.0401 (6)	0.080 (2)	
C38	0.5995 (11)	0.8116 (6)	0.1176 (6)	0.074 (2)	
H38A	0.6572	0.8033	0.1875	0.089*	
H38B	0.6887	0.7586	0.0887	0.089*	
C39	0.2545 (11)	0.9088 (6)	0.1149 (6)	0.0697 (18)	
H39A	0.1074	0.9139	0.1019	0.084*	
H39B	0.2708	0.9288	0.1786	0.084*	
C40	0.2126 (13)	0.3628 (7)	0.2935 (7)	0.099 (3)	
H40A	0.0770	0.4090	0.2924	0.148*	
H40B	0.2939	0.3595	0.3542	0.148*	
H40C	0.1986	0.2858	0.3007	0.148*	
C41	0.6102 (10)	0.5876 (6)	0.0341 (6)	0.0737 (18)	
H41A	0.6483	0.6416	−0.0332	0.111*	
H41B	0.6395	0.5109	0.0311	0.111*	
H41C	0.6889	0.5893	0.0934	0.111*	
C42	0.2981 (15)	1.1158 (6)	−0.0020 (8)	0.107 (3)	
H42A	0.3762	1.1567	−0.0620	0.161*	
H42B	0.3340	1.1281	0.0626	0.161*	
H42C	0.1517	1.1440	−0.0184	0.161*	
C43	0.7800 (16)	0.2039 (9)	0.1625 (10)	0.136 (4)	
H43A	0.8744	0.2396	0.1892	0.163*	
H43B	0.8278	0.2000	0.0927	0.163*	
C44	0.7712 (17)	0.0848 (11)	0.2426 (11)	0.152 (4)	
H44A	0.7401	0.0891	0.3134	0.228*	
H44B	0.9035	0.0347	0.2460	0.228*	
H44C	0.6646	0.0546	0.2196	0.228*	

### Atomic displacement parameters (Å^2^)

**Table:**

	*U*^11^	*U*^22^	*U*^33^	*U*^12^	*U*^13^	*U*^23^
O1	0.058 (3)	0.067 (3)	0.084 (3)	0.002 (2)	0.002 (2)	−0.010 (3)
O2	0.085 (4)	0.084 (3)	0.088 (4)	−0.005 (3)	0.036 (3)	−0.019 (3)
O3	0.073 (4)	0.108 (4)	0.167 (6)	−0.031 (3)	0.005 (4)	−0.058 (4)
O4	0.072 (4)	0.091 (4)	0.158 (6)	−0.001 (3)	−0.011 (3)	−0.050 (4)
O5	0.070 (3)	0.099 (4)	0.142 (5)	0.009 (3)	−0.041 (3)	−0.038 (3)
O6	0.073 (4)	0.125 (5)	0.158 (6)	−0.036 (4)	0.008 (4)	−0.038 (4)
C1	0.052 (4)	0.051 (4)	0.068 (5)	0.001 (3)	0.000 (3)	−0.030 (3)
C2	0.049 (4)	0.054 (4)	0.056 (4)	−0.006 (3)	−0.006 (3)	−0.019 (3)
C3	0.053 (4)	0.053 (4)	0.071 (5)	−0.008 (3)	0.005 (3)	−0.013 (3)
C4	0.071 (4)	0.070 (4)	0.056 (4)	−0.013 (3)	0.015 (3)	−0.011 (3)
C5	0.064 (4)	0.066 (4)	0.051 (4)	−0.007 (3)	0.009 (3)	−0.019 (3)
C6	0.053 (4)	0.060 (4)	0.044 (4)	−0.007 (3)	0.001 (3)	−0.020 (3)
C7	0.052 (4)	0.055 (4)	0.047 (4)	−0.005 (3)	−0.006 (3)	−0.016 (3)
C8	0.070 (4)	0.068 (4)	0.045 (4)	−0.008 (3)	0.002 (3)	−0.022 (3)
C9	0.081 (5)	0.066 (4)	0.057 (4)	−0.009 (3)	−0.001 (3)	−0.016 (3)
C10	0.057 (4)	0.055 (4)	0.062 (4)	−0.009 (3)	−0.003 (3)	−0.018 (3)
C11	0.048 (3)	0.060 (4)	0.055 (4)	−0.009 (3)	0.003 (3)	−0.024 (3)
C12	0.061 (4)	0.065 (4)	0.063 (4)	−0.006 (3)	0.003 (3)	−0.032 (3)
C13	0.077 (5)	0.076 (4)	0.088 (5)	−0.015 (3)	0.000 (4)	−0.039 (4)
C14	0.063 (4)	0.062 (4)	0.083 (5)	−0.014 (3)	0.001 (4)	−0.027 (4)
C15	0.057 (4)	0.070 (4)	0.101 (6)	−0.021 (3)	0.001 (4)	−0.022 (4)
C16	0.057 (4)	0.071 (4)	0.085 (5)	−0.011 (3)	0.010 (3)	−0.027 (4)
C17	0.064 (4)	0.061 (4)	0.076 (5)	−0.009 (3)	−0.004 (3)	−0.014 (3)
C18	0.067 (4)	0.071 (4)	0.091 (5)	−0.010 (3)	−0.020 (4)	−0.029 (4)
C19	0.058 (4)	0.064 (4)	0.059 (4)	−0.011 (3)	−0.006 (3)	−0.023 (3)
C20	0.092 (6)	0.069 (5)	0.142 (8)	−0.019 (4)	0.002 (5)	−0.041 (5)
C21	0.064 (5)	0.086 (5)	0.106 (6)	0.008 (4)	−0.003 (4)	−0.029 (5)
C22	0.108 (8)	0.145 (9)	0.155 (11)	0.053 (7)	0.023 (7)	0.023 (8)
C23	0.061 (5)	0.077 (5)	0.097 (6)	−0.012 (4)	−0.007 (4)	−0.022 (4)
C24	0.060 (4)	0.071 (5)	0.085 (5)	−0.015 (3)	0.000 (4)	−0.025 (4)
C25	0.066 (5)	0.087 (5)	0.103 (6)	−0.017 (4)	−0.012 (4)	−0.038 (5)
C26	0.081 (5)	0.097 (6)	0.081 (6)	−0.020 (4)	−0.016 (4)	−0.041 (4)
C27	0.079 (5)	0.088 (5)	0.059 (4)	−0.019 (4)	−0.012 (3)	−0.025 (4)
C28	0.051 (4)	0.065 (4)	0.054 (4)	−0.010 (3)	−0.008 (3)	−0.020 (3)
C29	0.056 (4)	0.074 (5)	0.060 (4)	−0.009 (3)	−0.004 (3)	−0.020 (3)
C30	0.078 (5)	0.077 (5)	0.049 (4)	−0.010 (3)	0.000 (3)	−0.016 (3)
C31	0.088 (5)	0.077 (5)	0.059 (5)	−0.010 (4)	−0.004 (4)	−0.022 (4)
C32	0.062 (4)	0.070 (4)	0.050 (4)	−0.004 (3)	−0.005 (3)	−0.019 (3)
C33	0.050 (4)	0.069 (4)	0.044 (4)	−0.005 (3)	−0.004 (3)	−0.017 (3)
C34	0.074 (5)	0.080 (5)	0.059 (4)	−0.015 (4)	−0.007 (3)	−0.016 (3)
C35	0.079 (5)	0.076 (5)	0.066 (5)	−0.006 (4)	−0.001 (4)	−0.006 (4)
C36	0.068 (5)	0.070 (5)	0.078 (5)	−0.014 (3)	−0.005 (4)	−0.016 (4)
C37	0.066 (5)	0.089 (5)	0.084 (5)	−0.021 (4)	0.000 (4)	−0.026 (4)
C38	0.067 (5)	0.082 (5)	0.072 (5)	−0.011 (4)	−0.013 (4)	−0.027 (4)
C39	0.068 (4)	0.072 (4)	0.065 (4)	−0.002 (3)	−0.003 (3)	−0.024 (4)
C40	0.088 (6)	0.083 (5)	0.104 (7)	−0.024 (4)	0.024 (5)	−0.006 (5)
C41	0.056 (4)	0.093 (5)	0.069 (4)	−0.003 (3)	0.006 (3)	−0.031 (4)
C42	0.115 (7)	0.075 (6)	0.122 (7)	−0.014 (5)	−0.005 (6)	−0.025 (5)
C43	0.094 (7)	0.109 (8)	0.172 (11)	0.015 (6)	0.010 (7)	−0.027 (8)
C44	0.100 (8)	0.138 (10)	0.179 (11)	0.021 (6)	0.000 (7)	−0.031 (9)

### Geometric parameters (Å, °)

**Table:**

O1—C1	1.314 (7)	C21—H21B	0.9700
O1—C21	1.434 (8)	C22—H22A	0.9600
O2—C1	1.189 (8)	C22—H22B	0.9600
O3—C15	1.213 (7)	C22—H22C	0.9600
O4—C23	1.360 (10)	C23—C24	1.518 (10)
O4—C43	1.445 (11)	C24—C25	1.538 (9)
O5—C23	1.171 (8)	C24—C29	1.539 (9)
O6—C37	1.188 (8)	C24—C40	1.547 (11)
C1—C2	1.551 (8)	C25—C26	1.497 (11)
C2—C3	1.526 (9)	C25—H25A	0.9700
C2—C18	1.545 (8)	C25—H25B	0.9700
C2—C7	1.562 (8)	C26—C27	1.504 (10)
C3—C4	1.514 (9)	C26—H26A	0.9700
C3—H3A	0.9700	C26—H26B	0.9700
C3—H3B	0.9700	C27—C28	1.554 (8)
C4—C5	1.519 (9)	C27—H27A	0.9700
C4—H4A	0.9700	C27—H27B	0.9700
C4—H4B	0.9700	C28—C41	1.530 (9)
C5—C6	1.528 (9)	C28—C29	1.551 (8)
C5—H5A	0.9700	C28—C33	1.561 (8)
C5—H5B	0.9700	C29—C30	1.530 (8)
C6—C19	1.545 (8)	C29—H29	0.9800
C6—C7	1.546 (8)	C30—C31	1.501 (9)
C6—C11	1.578 (8)	C30—H30A	0.9700
C7—C8	1.512 (8)	C30—H30B	0.9700
C7—H7	0.9800	C31—C32	1.541 (9)
C8—C9	1.524 (8)	C31—H31A	0.9700
C8—H8A	0.9700	C31—H31B	0.9700
C8—H8B	0.9700	C32—C38	1.524 (9)
C9—C10	1.508 (8)	C32—C39	1.526 (9)
C9—H9A	0.9700	C32—C33	1.553 (8)
C9—H9B	0.9700	C33—C34	1.510 (9)
C10—C11	1.524 (8)	C33—H33	0.9800
C10—C17	1.529 (9)	C34—C35	1.527 (10)
C10—C16	1.551 (9)	C34—H34A	0.9700
C11—C12	1.515 (8)	C34—H34B	0.9700
C11—H11	0.9800	C35—C36	1.525 (9)
C12—C13	1.531 (9)	C35—H35A	0.9700
C12—H12A	0.9700	C35—H35B	0.9700
C12—H12B	0.9700	C36—C39	1.504 (10)
C13—C14	1.521 (10)	C36—C37	1.519 (10)
C13—H13A	0.9700	C36—C42	1.527 (10)
C13—H13B	0.9700	C37—C38	1.492 (10)
C14—C15	1.510 (10)	C38—H38A	0.9700
C14—C17	1.519 (9)	C38—H38B	0.9700
C14—C20	1.528 (9)	C39—H39A	0.9700
C15—C16	1.497 (10)	C39—H39B	0.9700
C16—H16A	0.9700	C40—H40A	0.9600
C16—H16B	0.9700	C40—H40B	0.9600
C17—H17A	0.9700	C40—H40C	0.9600
C17—H17B	0.9700	C41—H41A	0.9600
C18—H18A	0.9600	C41—H41B	0.9600
C18—H18B	0.9600	C41—H41C	0.9600
C18—H18C	0.9600	C42—H42A	0.9600
C19—H19A	0.9600	C42—H42B	0.9600
C19—H19B	0.9600	C42—H42C	0.9600
C19—H19C	0.9600	C43—C44	1.502 (15)
C20—H20A	0.9600	C43—H43A	0.9700
C20—H20B	0.9600	C43—H43B	0.9700
C20—H20C	0.9600	C44—H44A	0.9600
C21—C22	1.483 (12)	C44—H44B	0.9600
C21—H21A	0.9700	C44—H44C	0.9600
			
C1—O1—C21	113.3 (5)	H22B—C22—H22C	109.5
C23—O4—C43	113.4 (7)	O5—C23—O4	121.7 (7)
O2—C1—O1	123.4 (5)	O5—C23—C24	126.8 (8)
O2—C1—C2	123.7 (6)	O4—C23—C24	111.5 (6)
O1—C1—C2	112.7 (6)	C23—C24—C25	113.1 (7)
C3—C2—C18	107.0 (5)	C23—C24—C29	113.9 (6)
C3—C2—C1	112.5 (5)	C25—C24—C29	108.1 (6)
C18—C2—C1	103.7 (5)	C23—C24—C40	105.0 (6)
C3—C2—C7	110.3 (5)	C25—C24—C40	107.6 (6)
C18—C2—C7	109.8 (4)	C29—C24—C40	108.9 (6)
C1—C2—C7	113.1 (5)	C26—C25—C24	114.1 (6)
C4—C3—C2	113.0 (5)	C26—C25—H25A	108.7
C4—C3—H3A	109.0	C24—C25—H25A	108.7
C2—C3—H3A	109.0	C26—C25—H25B	108.7
C4—C3—H3B	109.0	C24—C25—H25B	108.7
C2—C3—H3B	109.0	H25A—C25—H25B	107.6
H3A—C3—H3B	107.8	C25—C26—C27	111.2 (7)
C3—C4—C5	111.9 (5)	C25—C26—H26A	109.4
C3—C4—H4A	109.2	C27—C26—H26A	109.4
C5—C4—H4A	109.2	C25—C26—H26B	109.4
C3—C4—H4B	109.2	C27—C26—H26B	109.4
C5—C4—H4B	109.2	H26A—C26—H26B	108.0
H4A—C4—H4B	107.9	C26—C27—C28	113.9 (5)
C4—C5—C6	113.6 (5)	C26—C27—H27A	108.8
C4—C5—H5A	108.8	C28—C27—H27A	108.8
C6—C5—H5A	108.8	C26—C27—H27B	108.8
C4—C5—H5B	108.8	C28—C27—H27B	108.8
C6—C5—H5B	108.8	H27A—C27—H27B	107.7
H5A—C5—H5B	107.7	C41—C28—C29	112.3 (5)
C5—C6—C19	107.6 (4)	C41—C28—C27	107.7 (6)
C5—C6—C7	110.2 (5)	C29—C28—C27	108.3 (5)
C19—C6—C7	110.9 (5)	C41—C28—C33	113.1 (5)
C5—C6—C11	108.6 (5)	C29—C28—C33	108.0 (5)
C19—C6—C11	113.1 (5)	C27—C28—C33	107.2 (5)
C7—C6—C11	106.4 (4)	C30—C29—C24	116.3 (5)
C8—C7—C6	112.8 (5)	C30—C29—C28	111.0 (5)
C8—C7—C2	115.1 (5)	C24—C29—C28	115.7 (6)
C6—C7—C2	114.0 (4)	C30—C29—H29	104.0
C8—C7—H7	104.5	C24—C29—H29	104.0
C6—C7—H7	104.5	C28—C29—H29	104.0
C2—C7—H7	104.5	C31—C30—C29	110.2 (5)
C7—C8—C9	108.5 (5)	C31—C30—H30A	109.6
C7—C8—H8A	110.0	C29—C30—H30A	109.6
C9—C8—H8A	110.0	C31—C30—H30B	109.6
C7—C8—H8B	110.0	C29—C30—H30B	109.6
C9—C8—H8B	110.0	H30A—C30—H30B	108.1
H8A—C8—H8B	108.4	C30—C31—C32	115.1 (6)
C10—C9—C8	115.1 (5)	C30—C31—H31A	108.5
C10—C9—H9A	108.5	C32—C31—H31A	108.5
C8—C9—H9A	108.5	C30—C31—H31B	108.5
C10—C9—H9B	108.5	C32—C31—H31B	108.5
C8—C9—H9B	108.5	H31A—C31—H31B	107.5
H9A—C9—H9B	107.5	C38—C32—C39	100.6 (5)
C9—C10—C11	110.9 (5)	C38—C32—C31	115.8 (5)
C9—C10—C17	110.4 (5)	C39—C32—C31	109.4 (6)
C11—C10—C17	109.2 (5)	C38—C32—C33	113.5 (5)
C9—C10—C16	114.7 (6)	C39—C32—C33	108.1 (5)
C11—C10—C16	112.0 (5)	C31—C32—C33	109.0 (5)
C17—C10—C16	98.9 (5)	C34—C33—C32	108.7 (5)
C12—C11—C10	110.9 (5)	C34—C33—C28	114.8 (5)
C12—C11—C6	113.4 (4)	C32—C33—C28	116.1 (4)
C10—C11—C6	116.3 (5)	C34—C33—H33	105.4
C12—C11—H11	105.0	C32—C33—H33	105.4
C10—C11—H11	105.0	C28—C33—H33	105.4
C6—C11—H11	105.0	C33—C34—C35	113.5 (6)
C11—C12—C13	112.2 (5)	C33—C34—H34A	108.9
C11—C12—H12A	109.2	C35—C34—H34A	108.9
C13—C12—H12A	109.2	C33—C34—H34B	108.9
C11—C12—H12B	109.2	C35—C34—H34B	108.9
C13—C12—H12B	109.2	H34A—C34—H34B	107.7
H12A—C12—H12B	107.9	C36—C35—C34	112.5 (5)
C14—C13—C12	112.5 (6)	C36—C35—H35A	109.1
C14—C13—H13A	109.1	C34—C35—H35A	109.1
C12—C13—H13A	109.1	C36—C35—H35B	109.1
C14—C13—H13B	109.1	C34—C35—H35B	109.1
C12—C13—H13B	109.1	H35A—C35—H35B	107.8
H13A—C13—H13B	107.8	C39—C36—C37	100.8 (6)
C15—C14—C17	100.1 (6)	C39—C36—C35	108.0 (5)
C15—C14—C13	106.8 (5)	C37—C36—C35	106.2 (6)
C17—C14—C13	107.6 (5)	C39—C36—C42	116.0 (7)
C15—C14—C20	114.4 (6)	C37—C36—C42	113.8 (6)
C17—C14—C20	114.5 (6)	C35—C36—C42	111.2 (6)
C13—C14—C20	112.5 (6)	O6—C37—C38	125.7 (7)
O3—C15—C16	124.3 (6)	O6—C37—C36	125.5 (7)
O3—C15—C14	126.1 (7)	C38—C37—C36	108.8 (6)
C16—C15—C14	109.6 (5)	C37—C38—C32	104.8 (5)
C15—C16—C10	104.6 (5)	C37—C38—H38A	110.8
C15—C16—H16A	110.8	C32—C38—H38A	110.8
C10—C16—H16A	110.8	C37—C38—H38B	110.8
C15—C16—H16B	110.8	C32—C38—H38B	110.8
C10—C16—H16B	110.8	H38A—C38—H38B	108.9
H16A—C16—H16B	108.9	C36—C39—C32	103.4 (6)
C14—C17—C10	104.1 (5)	C36—C39—H39A	111.1
C14—C17—H17A	110.9	C32—C39—H39A	111.1
C10—C17—H17A	110.9	C36—C39—H39B	111.1
C14—C17—H17B	110.9	C32—C39—H39B	111.1
C10—C17—H17B	110.9	H39A—C39—H39B	109.0
H17A—C17—H17B	109.0	C24—C40—H40A	109.5
C2—C18—H18A	109.5	C24—C40—H40B	109.5
C2—C18—H18B	109.5	H40A—C40—H40B	109.5
H18A—C18—H18B	109.5	C24—C40—H40C	109.5
C2—C18—H18C	109.5	H40A—C40—H40C	109.5
H18A—C18—H18C	109.5	H40B—C40—H40C	109.5
H18B—C18—H18C	109.5	C28—C41—H41A	109.5
C6—C19—H19A	109.5	C28—C41—H41B	109.5
C6—C19—H19B	109.5	H41A—C41—H41B	109.5
H19A—C19—H19B	109.5	C28—C41—H41C	109.5
C6—C19—H19C	109.5	H41A—C41—H41C	109.5
H19A—C19—H19C	109.5	H41B—C41—H41C	109.5
H19B—C19—H19C	109.5	C36—C42—H42A	109.5
C14—C20—H20A	109.5	C36—C42—H42B	109.5
C14—C20—H20B	109.5	H42A—C42—H42B	109.5
H20A—C20—H20B	109.5	C36—C42—H42C	109.5
C14—C20—H20C	109.5	H42A—C42—H42C	109.5
H20A—C20—H20C	109.5	H42B—C42—H42C	109.5
H20B—C20—H20C	109.5	O4—C43—C44	106.1 (8)
O1—C21—C22	107.3 (7)	O4—C43—H43A	110.5
O1—C21—H21A	110.3	C44—C43—H43A	110.5
C22—C21—H21A	110.3	O4—C43—H43B	110.5
O1—C21—H21B	110.2	C44—C43—H43B	110.5
C22—C21—H21B	110.2	H43A—C43—H43B	108.7
H21A—C21—H21B	108.5	C43—C44—H44A	109.5
C21—C22—H22A	109.5	C43—C44—H44B	109.5
C21—C22—H22B	109.5	H44A—C44—H44B	109.5
H22A—C22—H22B	109.5	C43—C44—H44C	109.5
C21—C22—H22C	109.5	H44A—C44—H44C	109.5
H22A—C22—H22C	109.5	H44B—C44—H44C	109.5
			
C21—O1—C1—O2	−3.1 (9)	C43—O4—C23—O5	−1.3 (12)
C21—O1—C1—C2	−178.3 (5)	C43—O4—C23—C24	178.2 (7)
O2—C1—C2—C3	174.0 (6)	O5—C23—C24—C25	163.8 (8)
O1—C1—C2—C3	−10.7 (7)	O4—C23—C24—C25	−15.7 (9)
O2—C1—C2—C18	−70.7 (7)	O5—C23—C24—C29	39.9 (11)
O1—C1—C2—C18	104.5 (6)	O4—C23—C24—C29	−139.6 (7)
O2—C1—C2—C7	48.2 (8)	O5—C23—C24—C40	−79.1 (10)
O1—C1—C2—C7	−136.6 (5)	O4—C23—C24—C40	101.4 (7)
C18—C2—C3—C4	172.0 (5)	C23—C24—C25—C26	−73.0 (8)
C1—C2—C3—C4	−74.7 (6)	C29—C24—C25—C26	54.0 (9)
C7—C2—C3—C4	52.6 (6)	C40—C24—C25—C26	171.4 (6)
C2—C3—C4—C5	−54.8 (7)	C24—C25—C26—C27	−56.5 (9)
C3—C4—C5—C6	54.5 (7)	C25—C26—C27—C28	55.0 (8)
C4—C5—C6—C19	69.2 (6)	C26—C27—C28—C41	70.2 (8)
C4—C5—C6—C7	−51.9 (6)	C26—C27—C28—C29	−51.4 (8)
C4—C5—C6—C11	−168.0 (5)	C26—C27—C28—C33	−167.8 (6)
C5—C6—C7—C8	−175.6 (5)	C23—C24—C29—C30	−59.2 (9)
C19—C6—C7—C8	65.4 (6)	C25—C24—C29—C30	174.2 (6)
C11—C6—C7—C8	−58.0 (6)	C40—C24—C29—C30	57.6 (8)
C5—C6—C7—C2	50.7 (6)	C23—C24—C29—C28	73.9 (8)
C19—C6—C7—C2	−68.4 (6)	C25—C24—C29—C28	−52.7 (7)
C11—C6—C7—C2	168.3 (5)	C40—C24—C29—C28	−169.3 (5)
C3—C2—C7—C8	176.0 (5)	C41—C28—C29—C30	68.4 (7)
C18—C2—C7—C8	58.3 (7)	C27—C28—C29—C30	−172.9 (5)
C1—C2—C7—C8	−57.0 (6)	C33—C28—C29—C30	−57.1 (6)
C3—C2—C7—C6	−51.4 (6)	C41—C28—C29—C24	−67.1 (7)
C18—C2—C7—C6	−169.1 (5)	C27—C28—C29—C24	51.6 (7)
C1—C2—C7—C6	75.6 (7)	C33—C28—C29—C24	167.4 (5)
C6—C7—C8—C9	61.7 (6)	C24—C29—C30—C31	−164.2 (6)
C2—C7—C8—C9	−165.1 (5)	C28—C29—C30—C31	60.6 (7)
C7—C8—C9—C10	−56.3 (7)	C29—C30—C31—C32	−57.4 (8)
C8—C9—C10—C11	48.9 (7)	C30—C31—C32—C38	−79.5 (8)
C8—C9—C10—C17	170.1 (6)	C30—C31—C32—C39	167.8 (5)
C8—C9—C10—C16	−79.2 (7)	C30—C31—C32—C33	49.7 (7)
C9—C10—C11—C12	−178.5 (5)	C38—C32—C33—C34	−48.7 (7)
C17—C10—C11—C12	59.6 (6)	C39—C32—C33—C34	61.9 (7)
C16—C10—C11—C12	−48.9 (6)	C31—C32—C33—C34	−179.2 (5)
C9—C10—C11—C6	−47.0 (7)	C38—C32—C33—C28	82.6 (7)
C17—C10—C11—C6	−168.9 (5)	C39—C32—C33—C28	−166.8 (5)
C16—C10—C11—C6	82.6 (6)	C31—C32—C33—C28	−47.9 (7)
C5—C6—C11—C12	−60.3 (6)	C41—C28—C33—C34	55.9 (7)
C19—C6—C11—C12	59.1 (7)	C29—C28—C33—C34	−179.2 (5)
C7—C6—C11—C12	−178.9 (5)	C27—C28—C33—C34	−62.6 (7)
C5—C6—C11—C10	169.4 (5)	C41—C28—C33—C32	−72.4 (7)
C19—C6—C11—C10	−71.2 (6)	C29—C28—C33—C32	52.5 (6)
C7—C6—C11—C10	50.8 (6)	C27—C28—C33—C32	169.0 (5)
C10—C11—C12—C13	−47.5 (7)	C32—C33—C34—C35	−48.0 (7)
C6—C11—C12—C13	179.5 (6)	C28—C33—C34—C35	−179.9 (5)
C11—C12—C13—C14	48.3 (7)	C33—C34—C35—C36	47.0 (8)
C12—C13—C14—C15	47.1 (7)	C34—C35—C36—C39	−57.7 (8)
C12—C13—C14—C17	−59.6 (7)	C34—C35—C36—C37	49.7 (8)
C12—C13—C14—C20	173.4 (5)	C34—C35—C36—C42	174.0 (7)
C17—C14—C15—O3	−158.8 (8)	C39—C36—C37—O6	−158.6 (8)
C13—C14—C15—O3	89.2 (8)	C35—C36—C37—O6	88.9 (9)
C20—C14—C15—O3	−35.9 (11)	C42—C36—C37—O6	−33.8 (12)
C17—C14—C15—C16	21.1 (7)	C39—C36—C37—C38	21.6 (8)
C13—C14—C15—C16	−90.9 (7)	C35—C36—C37—C38	−90.9 (7)
C20—C14—C15—C16	144.0 (7)	C42—C36—C37—C38	146.5 (7)
O3—C15—C16—C10	−172.4 (7)	O6—C37—C38—C32	−173.1 (8)
C14—C15—C16—C10	7.7 (8)	C36—C37—C38—C32	6.6 (8)
C9—C10—C16—C15	−150.3 (5)	C39—C32—C38—C37	−31.7 (7)
C11—C10—C16—C15	82.1 (6)	C31—C32—C38—C37	−149.5 (6)
C17—C10—C16—C15	−32.9 (7)	C33—C32—C38—C37	83.4 (6)
C15—C14—C17—C10	−42.7 (6)	C37—C36—C39—C32	−41.8 (7)
C13—C14—C17—C10	68.6 (7)	C35—C36—C39—C32	69.4 (6)
C20—C14—C17—C10	−165.6 (6)	C42—C36—C39—C32	−165.1 (6)
C9—C10—C17—C14	168.1 (6)	C38—C32—C39—C36	46.3 (6)
C11—C10—C17—C14	−69.7 (6)	C31—C32—C39—C36	168.7 (5)
C16—C10—C17—C14	47.4 (7)	C33—C32—C39—C36	−72.8 (6)
C1—O1—C21—C22	−156.5 (8)	C23—O4—C43—C44	105.9 (10)

### Table 1 Comparison of the puckering parameters (Å, °) for the six and five-membered rings in molecules 1 and 2

**Table:**

Molecule		1			2	
Puckering parameters	Q	θ	φ	Q	θ	φ
Ring *A*1, *A*2	0.536 (7)	180.0 (7)	273 (27)	0.545 (8)	177.2 (8)	251 (29)
Ring *B*1, *B*2	0.557 (7)	9.5 (7)	72 (4)	0.561 (7)	7.8 (7)	71 (5)
Ring *C*1, *C*2	0.633 (7)	164.8 (6)	120 (3)	0.646 (8)	163.2 (7)	131 (2)
Puckering parameters		q_2_	φ_2_		q_2_	φ_2_
Ring *D*1, *D*2		0.467 (8)	333.7 (10)		0.452 (8)	332.2 (11)

Ring *A*1 atoms C2–C7, *A*2 C24–C29, *B*1 C6–C11, *B*2 C28–C33, *C*1 C10–C14/C17, *C*2 C32–C36/C39, *D*1 C14–C16/C10/C17, and *D*2 C36–C38/C32/C39.
